# Multidrug resistant tuberculosis co-existing with aspergilloma and invasive aspergillosis in a 50 year old diabetic woman: a case report

**DOI:** 10.1186/1757-1626-1-303

**Published:** 2008-11-08

**Authors:** Anita A Kumar, Ghanshyam Palamaner Subash Shantha, Vijay Jeyachandran, K Rajkumar, Senthilkumar Natesan, Devasena Srinivasan, Leena Dennis Joseph, Manjunath Sundaresan, Deepan Rajamanickam

**Affiliations:** 1 Department of General Medicine, Sri Ramachandra University, Chennai, India; 2 Department of Pathology, Sri Ramachandra University, Chennai, India

## Abstract

Aspergilloma and invasive aspergillosis coexisting with multidrug resistant Mycobacterium tuberculosis (MDR-TB) in the same patient is a rare entity. We report a 50 year old South Indian woman, a diabetic, who presented to us with complaints of productive cough and hemoptysis for the past 2 months. She was diagnosed to have pulmonary tuberculosis 2 years ago for which she took irregular treatment. Lung imaging showed features of a thick walled cavity in the right upper lobe with an indwelling aspergilloma. She underwent a right lung upper lobe resection. Biopsy and culture of the resected specimen showed the coexistence of Aspergillus fumigatus and multi-drug resistant Mycobacterium tuberculosis. 2 blood cultures grew Aspergillus fumigatus. She was successfully treated with Voriconazole and anti tuberculous therapy against MDR-TB.

## Background

Pulmonary tuberculosis is the most commonly associated disease in cases of secondary aspergilloma [1]. Generally aspergilloma is seen residing in an old tuberculous cavity. In this case report we present a rare case of an aspergilloma co-existing with multidrug resistant mycobacterium tuberculous in an old cavity. This patient also had invasive aspergillosis. This combination is uncommon and to the best of our knowledge is not reported in literature.

## Case presentation

**Figure 1:**
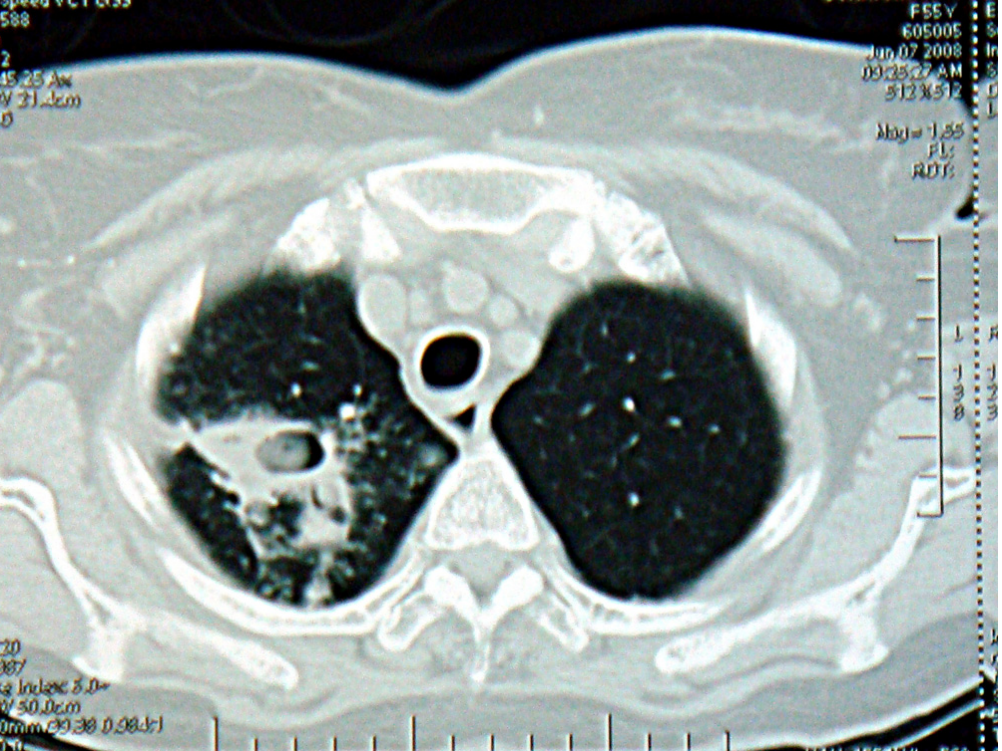
**Computed tomogram of the thorax**. Thick walled cavity in the right lung upper lobe with an indwelling aspergilloma.

**Figure 2:**
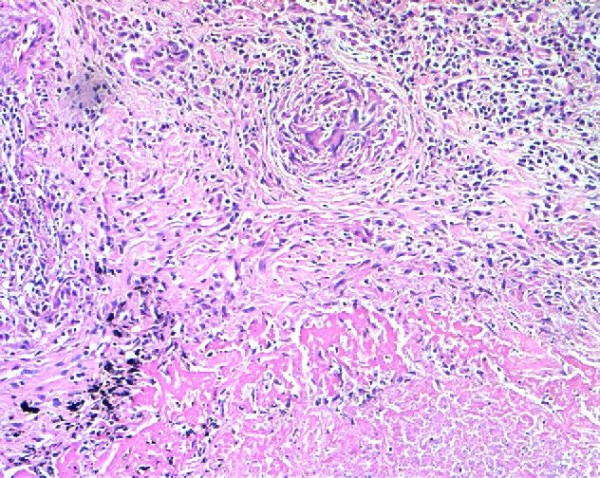
**Caseating granuloma suggestive of Tubercular infection**. Histology picture showing Langhan's giant cells, epitheloid cells with surrounding necrosis (H and E × 100).

**Figure 3:**
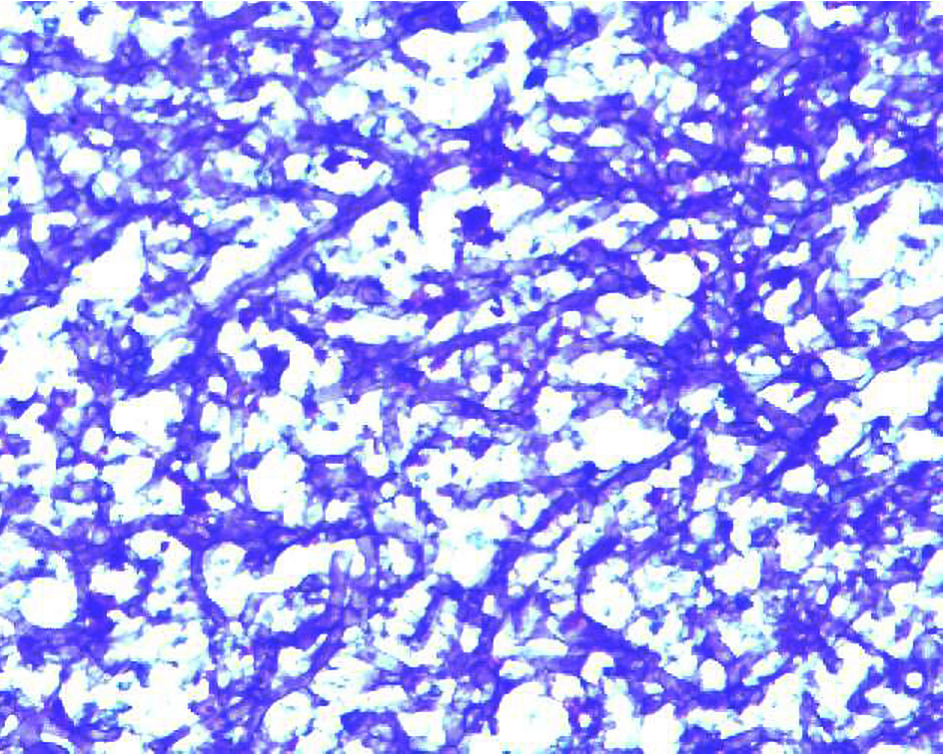
**Aspergillus species**. Histology picture showing fungal hyphae of Aspergillus (Periodic Acid Schiff-G stain × 400).

A 50 year old South Indian woman presented to the outpatient department of our tertiary care hospital with complaints of productive cough with hemoptysis for the past 2 months. She was a house wife and was from a low socio economic class. She was diagnosed to have pulmonary tuberculosis 2 years ago and was started on anti-tuberculous therapy consisting of Isoniazid (300 mg), Rifampicin (600 mg), Ethambutol (1200 mg), and Pyrazinamide (1500 mg). But within just 3 weeks she had discontinued treatment on her own. She suffered from diabetes mellitus with peripheral neuropathy for the past 8 years and was taking oral hypoglycemic agents for the same. There was no history of hypothyroidism, coronary artery disease, hepatic disease or renal disease. No history of relevant family diagnosis of parents, siblings, or children was elicited. She is not a known smoker or alcoholic. She is married and currently postmenopausal for past 5 years. She weighed 53 kgs and was 156 cms tall with a body mass index was 22 kg/m2. General physical examination was unremarkable. Respiratory system exam revealed bronchial breath sounds in the right infraclavicular area. Admission baseline investigations showed anemia. Renal and liver functions were within normal limits (Table 1). Computed tomogram of the thorax revealed a thick walled cavity in the right lung upper lobe with an indwelling aspergilloma (Figure 1). 3 sputum samples were tested positive for acid fast bacilli by Ziehl Neelsen's staining technique. Conventional method of culture on Lowenstein Jensen's medium yielded growth of M. tuberculosis in 6 weeks time. The anti-tuberculosis drug susceptibility performed by resistance ratio method using Lowenstein Jensen's medium showed resistance to Isoniazid, Rifampicin but sensitive to Ethambutol, Pyrazinamide and Streptomycin in their critical concentrations of 2 ug, 50 ug and 4 ug respectively as given by Lee and Heifet.8. Consequently in view of hemoptysis and presence of an aspergilloma a right upper lobectomy was performed (after anemia correction with 3 units of packed red cell transfusion). Biopsy of the resected specimen showed caseous necrosis and granuloma formation (Figure 2) and septate fungal elements suggestive of Aspergillus species (Figure 3). A fungal culture of the resected specimen in Sabouraud's dextrose agar grew 'dirty green colonies', with lactophenol cotton blue slide mount showed fungal elements characteristic of Aspergillus fumigatus. 2 Blood culture inoculated in Sabouraud's dextrose agar also grew Aspergillus fumigatus. In view of invasive Aspergillosis patient was given an oral loading dose of Voriconazole 400 mg 12^th ^hourly for 2 doses which was followed by an oral maintenance dose of 200 mg 12^th ^hourly was continued for 6 weeks. Initially before AFB culture reports were ready, the patient was initiated empirically on a daily dose of Isoniazid (300 mg), Ethambutol (1200 mg), Pyrazinamide (1500 mg) and Streptomycin 1 g. Rifampicin due to its interactions with Voriconazole was not included in the treatment regimen [2]. After culture demonstrated MDR-TB, Isoniazid was stopped and oral Levofloxacin 750 mg once a day and Ethionamide 250 mg 12^th ^hourly were included as per WHO protocol for MDR-TB [3]. Hemoptysis completely resolved after lobectomy. Within a week of initiating Voriconazole blood became sterile for fungal elements. After 3 weeks sputum became negative for acid fast bacilli. Currently patient is in the 4^th ^month of treatment and is doing well.

**Table 1:** Laboratory investigations

Hemoglobin 7 g/dl	
Total count	4,500 cells/mm3

Differential count	Poly morphs: 73%
	
	Lymphocytes: 24%
	
	Eosinophils: 3%

Platelet count	3,25,000 cells/mm3

Serum creatinine	0.9 mg/dl

Total serum bilirubin	1.1 mg/dl

Direct bilirubin	0.6 mg/dl

Random blood sugar	231 mg/dl

ELISA for HIV 1 and 2	non – reactive

Sputum AFB (3 samples)	Positive

Sputum culture	Mycobacterium tuberculosis (MDR-TB)

Blood culture (2 samples)	Aspergillus fumigatus

## Discussion

TB is principally a disease of poverty, with 95% of cases and 98% of deaths occurring in developing countries. Of these, more than half the cases occur in five South-East Asian countries [4]. Globally, about 3% of all newly diagnosed patients have MDR-TB [5]. Definition of multi-drug resistance refers to isolates resistant to both Isoniazid and Rifampicin with or without resistance to other drugs [5]. Three common forms of pulmonary disease associated with Aspergillus infection has been described, namely, allergic aspergillosis, colonizing aspergillosis, and invasive aspergillosis. A study showed that aspergilloma was commonly associated with pulmonary tuberculosis and affected the upper lobes in 94% of the cases [6]. Our patient also had pulmonary tuberculosis and aspergilloma had affected the right upper lobe. The uniqueness of our case report is that this association between MDR-TB, aspergilloma and invasive aspergillosis in the same patient is rare and to the best of our knowledge has not been reported in the literature before.

The natural history of aspergilloma is variable. Hemoptysis is the commonest mode of presentation, with an incidence of around 80%, which is life threatening in 30% [7]. In the majority of cases, the lesion remains stable, however, in approximately 10% of cases, it may decrease in size or resolve spontaneously without treatment [8]. Rarely, the aspergilloma increases in size [9]. Predicted mortality due to aspergilloma is reported at a rate of 6% per annum [10]. Surgery not only offers symptomatic control but also confers survival advantage [11]. Hemoptysis completely resolved in our patient after right lung upper lobe resection. Invasive aspergillosis is commonly seen in immunocompromised patients. Except for the poorly controlled diabetes, we could not identify any other risk factor for invasive fungal infection in our patient.

## Conclusion

Aspergilloma, invasive aspergillosis, and MDR-TB can coexist in the same patient. Hence patients who have a recurrence of tuberculosis, and who were previous defaulters of antitubercular therapy, an AFB culture should be performed in order to identify MDR-TB. Surgery often gives good results in the treatment of aspergilloma. Systemic antifungals should be administered against invasive fungal infections.

### Patient's perspective

I have realized my mistake of stopping the TB medicines. Now the treatment is prolonged and the injection is painful. I could have spread these resistant bacteria to many people. Hence forth I will be careful and follow my doctor's instructions. I have learnt my lesson for sure but in a hard way.

## Abbreviations

MDR-TB: Multi-Drug Resistant Tuberculosis; TB: Tuberculosis; AFB: Acid Fast Bacillus; WHO: World Health Organization.

## Competing interests

The authors declare that they have no competing interests.

## Authors' contributions

AAK, GPSS, VJ, RK, SN, DS, MS, DR were involved in the patient care. AAK and GPSS were also involved in acquisition of data, analysis and interpretation of data, review of literature, drafting and revising the manuscript. MS revised the manuscript for important intellectual content. LDJ was the pathologist involved in tissue analysis of the specimens. All authors read and approved the final manuscript.

## Consent

Written informed consent was obtained from the patient for publication of this case report and accompanying images. A copy of the written consent is available for review by the Editor-in-Chief of this journal.
